# Comparative Mitogenomics of Fungal Species in Stachybotryaceae Provides Evolutionary Insights into Hypocreales

**DOI:** 10.3390/ijms222413341

**Published:** 2021-12-12

**Authors:** Li-Yuan Ren, Shu Zhang, Yong-Jie Zhang

**Affiliations:** 1 School of Life Science, Shanxi University, Taiyuan 030006, China; 17835423798@163.com; 2 Key Laboratory of Microbial Resources Collection and Preservation, Ministry of Agriculture and Rural Affairs, Beijing 100081, China

**Keywords:** comparative mitogenomics, evolution, gene order, Hypocreales, mitochondrial genome, phylogeny, Stachybotryaceae, *Stachybotrys chartarum*

## Abstract

*Stachybotrys chartarum* is one of the world’s ten most feared fungi within the family Stachybotryaceae, although to date, not a single mitogenome has been documented for Stachybotryaceae. Herein, six mitogenomes of four different species in Stachybotryaceae are newly reported. The *S. chartarum* mitogenome was 30.7 kb in length and contained two introns (one each in *rnl* and *cox1*). A comparison of the mitogenomes of three different individuals of *S. chartarum* showed few nucleotide variations and conservation of gene content/order and intron insertion. A comparison of the mitogenomes of four different Stachybotryaceae species (*Memnoniella echinata*, *Myrothecium inundatum*, *S. chartarum*, and *S. chlorohalonata*), however, revealed variations in intron insertion, gene order/content, and *nad2/nad3* joining pattern. Further investigations on all Hypocreales species with available mitogenomes showed greater variabilities in gene order (six patterns) and *nad2/nad3* joining pattern (five patterns) although a dominant pattern always existed in each case. Ancestral state estimation showed that in each case the dominant pattern was always more ancestral than those rare patterns. Phylogenetic analyses based on mitochondrion-encoded genes supported the placement of Stachybotryaceae in Hypocreales. The crown age of Stachybotryaceae was estimated to be approximately the Early Cretaceous (141–142 Mya). This study greatly promotes our understanding of the evolution of fungal species in Hypocreales.

## 1. Introduction

Mitochondria, as energy factories, are essential organelles present in eukaryotic cells [1]. Mitochondria contain genetic materials called mitochondrial DNA (mtDNA) or mitochondrial genome (mitogenome). Fungi, as a large group of eukaryotic organisms, have mtDNA within their mitochondria in addition to the nuclear DNA present in the nucleus within their cells [2]. Over recent years, mitogenomes of an increasing number of fungal species have been sequenced. Fungal mitogenomes typically encode several common genes, including (1) genes encoding rRNAs (*rnl* for the large rRNA subunit and *rns* for the small rRNA subunit) and tRNAs (of almost all amino acids), and (2) genes related to protein subunits participating in oxidative phosphorylation (i.e., *cox1–3*, *cob*, *nad1–6* and *nad4L*) and ATP production (*atp6*, *8* and *9*) [3]. Different fungi, however, show dynamic mitogenomes concerning size and structures (e.g., number and length of introns, length of intergenic regions and repetitive elements) [4,5,6]. Due to the diversity at both intraspecific and interspecific levels, mtDNA has been used as an ideal marker for solving issues related to fungal phylogeny and evolution [5,7,8].

Stachybotryaceae is a fungal family within the order Hypocreales (Sordariomycetes, Ascomycota). With over 300 species described, this family includes important plant and human pathogens, as well as several species used in industrial and commercial applications as biodegraders and biocontrol agents [9]. According to morphology and multi-gene phylogeny, 39 genera are currently accepted in Stachybotryaceae, including *Memnoniella*, *Myrothecium*, and *Stachybotrys* (type genus of the family) [10]. Among these genera, *Stachybotrys* is the biggest, with over 100 described species (http://www.speciesfungorum.org/, accessed on 27 September 2021). Members in *Stachybotrys* are saprobes, commonly isolated from soil and decaying plant material, and are also associated with health risks in buildings with long-term water damage [11]. *Stachybotry schartarum*, a type of *Stachybotrys*, is known as “toxic black mold” in many places. The fungus is listed as one of “the world’s ten most feared fungi” because it can produce mycotoxins, result in serious building related illness, agricultural damage and a variety of symptoms in humans, and even cause the death of animals [12]. Several novel compounds are also isolated from this species [13,14].

Currently, a total of 303 genera and 14 families are accepted for species in Hypocreales [10]; however, mitogenomes from only 27 genera in 9 families are available in Hypocreales, even though Hypocreales has been the order with the most available mitogenomes (~120 as of November 2021) in the kingdom of Fungi. This under-representation in genomic studies is unexpected considering their high species diversity and ecological importance. Although nuclear genomes of two *Stachybotrys* species (*S. chartarum* and *S. chlorohalonata*) have been reported [15,16], not a single mitogenome for species in Stachybotryaceae is currently available. In this study, we assembled the mitogenomes of six individuals belonging to four different species in Stachybotryaceae, and performed comparisons of different mitogenomes from both intraspecific and interspecific perspectives. The purposes of this study are (1) to describe the first mitogenome of *S. chartarum*, one of the world’s most feared fungi, (2) to investigate intraspecific and interspecific variations in mitogenomes in Stachybotryaceae, (3) to understand the evolution of mitochondrial gene order in Hypocreales, and 4) to infer the phylogenetic position of Stachybotryaceae and its divergence time. We hypothesize that Stachybotryaceae shows mitogenomic diversity at both the intraspecific and interspecific level. This study will greatly enrich the available mitogenome information in Hypocreales and provide a valuable reference for understanding species evolution in Hypocreales.

## 2. Results

### 2.1. Organization of the S. chartarum Mitogenome

**Figure 1:**
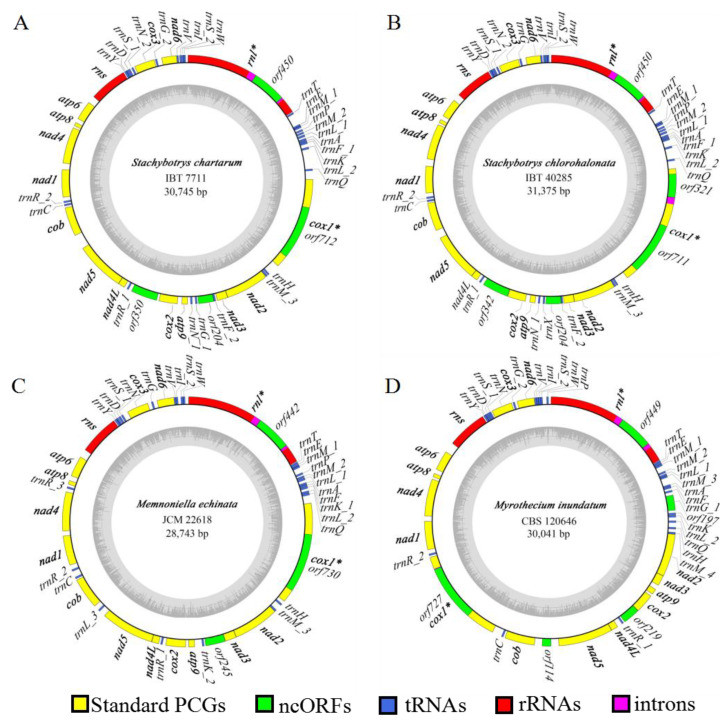
Circular map of mitogenomes of four Stachybotryaceae species. The outer ring marks relative positions of different genes, and the inner ring represents GC contents. All genes were transcribed at the same strand. Different kinds of genes/sequences are shown in different colors. The 14 core protein-encoding genes typically found in fungal mitogenomes are shown in bold. Intron-containing genes are followed by asterisks after gene names. ncORFs include both intronic and intergenic ORFs. Refer to Supplementary Table S1 for details of the annotations. (**A**) *S. chartarum*. (**B**) *S. chlorohalonata*. (**C**) *Mem. echinata*. (**D**) *Myr. inundatum*.

We chose the isolate IBT 7711 as a representative to describe the mitogenome of *S. chartarum*. Its mitogenome was a circular molecule of 30,745 bp with a high AT content of 73.7% (Figure 1A). It encoded 46 mitochondrial genes, including 16 protein-coding genes, two rRNA genes (*rnl* and *rns*), and 28 tRNA genes (*trn*). These protein-coding genes included 14 core protein-coding genes (PCGs, *nad1–6*, *4L*; *cob*; *cox1–3*; and *atp6*, *8*, *9*) typically found in fungal mitogenomes and two intergenic ORFs (i.e., *orf204* at the *nad3/atp9* intergenic region and *orf350* at the *cox2/nad4L* intergenic region) that encoded for hypothetical proteins. The 28 *trn* genes could accommodate for all 20 standard amino acids. They all displayed the classical cloverleaf structures apart from four (i.e., *trnL_1*, *trnL_2*, *trnS_1*, and *trnY*) having extra loops and *trnE*, which presented an anticodon loop with nine instead of the commonly observed seven nucleosides (Supplementary Figure S1). The lengths of these *trn* genes ranged from 71 bp to 85 bp. Among them, there were three *trn* genes for methionine with the same anticodons, two for glycine with the same anticodons, and two with different anticodons for each of arginine, asparagine, leucine, serine, and phenylalanine (Supplementary Table S1). All the mitochondrial genes were transcribed at the same strand. All protein-coding genes were started by ATG and terminated by TAA except *cob* and *cox3*, which were started by GTG and TTG, respectively, and *atp9* and *nad5*, which were terminated by TAG. Since proteins must begin with methionine, which only has one codon (i.e., ATG), *cob* and *cox3* are most likely post-transcriptionally modified to start with an A instead of G or T. The *S. chartarum* mitogenome was rather compact, with genic regions (with the inclusion of intronic regions) accounting for 82.1% and intergenic regions accounting for 17.9% of the overall mitogenome (Table 1).

Intronic regions had a total length of 3884 bp, accounting for 12.6% of the mitogenome. Two introns, one each into *rnl* and *cox1*, were identified (Figure 1A; Supplementary Table S2). Both introns belonged to the group I intron family, and the *rnl* intron (designated as mL2450 based on the nomenclature of Johansen and Haugen 2001)) and the *cox1* intron (designated as cox1P1125 based on the nomenclature of Zhang and Zhang 2019) belonged to subgroups IA and IB, respectively. Both introns conformed to typical nucleotide characteristics of group I introns with upstream exons ending with a base “T” and intron themselves ending with a base “G”. Each intron harbored an intronic ORF, coding for ribosomal protein S3 (*rps3*, *orf450* in *rnl*) or a LAGLIDADG homing endonuclease (*orf712* in *cox1*).

### 2.2. Comparison on Mitogenomes among Different Individuals of S. chartarum

Complete mitogenomes were successfully assembled for another two individuals (IBT 40288 and 40293) of *S. chartarum*. Comparison among the three individuals (IBT 7711, 40288, and 40293) showed that *S. chartarum* mitogenomes were rather conserved with weak size variations from 30,718 bp to 30,745 bp (Table 1). Although mitogenome sizes of IBT 7711 and 40293 happened to be identical (both at 30,745 bp), their sequences showed differences at 33 positions (including four alignment gaps and 29 variable sites) across the whole mitogenomes. Alignment of all the three mitogenomes revealed 33 sites with alignment gaps (i.e., indel sites) and 36 variable (polymorphic) sites, corresponding to a genetic divergence of 0.22%. These indel sites were mostly located at intergenic regions (24 out of 33), while these variable sites were mainly located at genic regions (23 out of 36). Variable sites at coding regions were all synonymous mutations, except for one nonsynonymous site which was found in each of the two intronic ORFs.

Consistent with the low intraspecific nucleotide variations, the three mitogenomes did not show variations in gene content and order, except that the intergenic *orf350* in IBT 7711 and 40293 had a 9 nt deletion in the middle of the sequence (thus renamed as *orf347* based on the length of encoded amino acid sequences) in IBT 40288 (Supplementary Table S1). Intron insertions and intron-encoded proteins were conserved among the three mitogenomes of *S. chartarum* (Supplementary Table S2).

### 2.3. Comparison on Mitogenomes among Different Species in Stachybotryaceae

**Figure 2:**
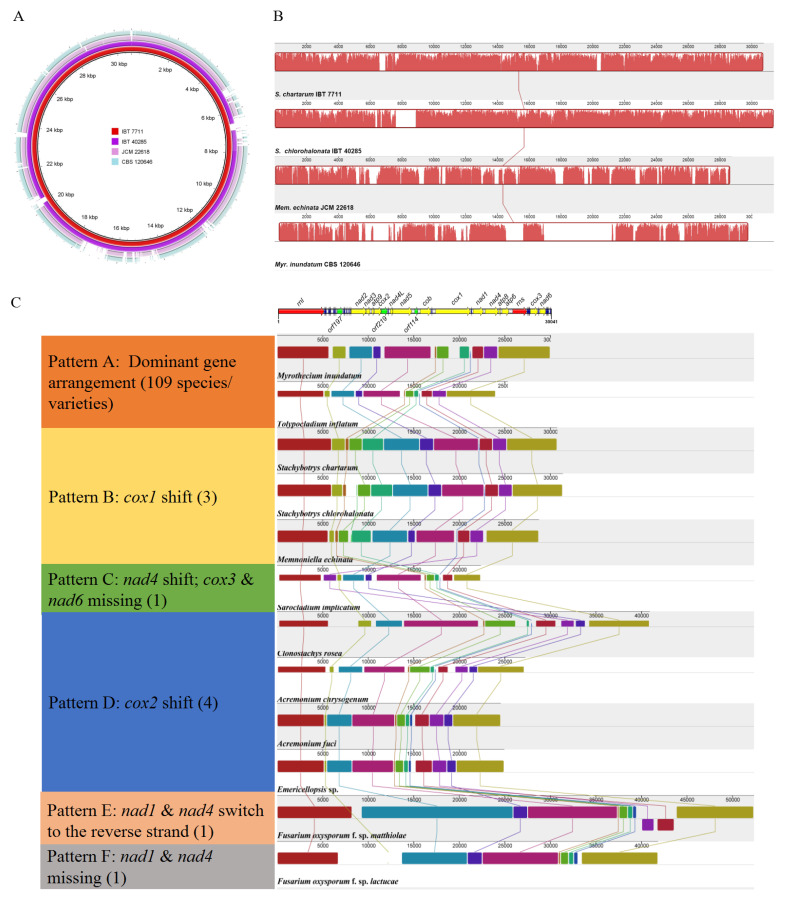
Alignment of mitogenomes of different Hypocreales species. (**A**) Alignment of four Stachybotryaceae species using BRIG. Each ring represents a mitogenome as shown by different colors. The mitogenome of *S. chartarum* IBT 7711 acts as the reference during alignment. (**B**) Alignment of four Stachybotryaceae species using Mauve. Corresponding color boxes are locally collinear blocks (LCBs). A sequence identity similarity profile is shown in each box. (**C**) Alignment of representative Hypocreales species using Mauve. Representative species for each kind of gene arrangement patterns were chosen to align. All mitogenomes were adjusted to a fixed starting point (i.e., 10 bp upstream of *rnl*). Mitochondrial gene order of *Myr. inundatum* is drawn on top of the figure. Notes are given on the left for each gene arrangement pattern relative to the dominant pattern. Each pattern is named with a different letter (A–F), and the number of mitogenomes of each pattern is given within parentheses. Refer to Supplementary Table S5 for more details of gene order for each species.

We further assembled complete mitogenomes of three other Stachybotryaceae species, viz. *Memnoniella echinata*, *Myrothecium inundatum*, and *Stachybotrys chlorohalonata*, and compared them with the mitogenome of *S. chartarum* IBT 7711. The four Stachybotryaceae species all had relatively small mitogenome sizes, ranging from 28.7 kb in *Mem. echinata* to 31.4 kb in *S. chlorohalonata* (Table 1). Comparative alignments of the four mitogenomes were carried out with two programs, BRIG and Mauve (Figure 2A,B). There was one homologous block among the four mitogenomes (Figure 2B), implying that the gene order or synteny of these mitogenomes was to a great extent conservative. Variations, however, were present with regard to gene content and order (Figure 1; Supplementary Table S1). Specifically, *S. chlorohalonata* showed almost identical gene content and order to its congeneric species *S. chartarum*, except that *trnG_1* and the intergenic *orf350* in the latter species turned into *trnX* (encoding tRNA molecule that linked to an undetermined amino acid with the unusual anticodon TCCC) and *orf342* (homologous to *orf350*), respectively, in the former species. Compared with *S. chartarum*, *Mem. echinata* contained only one intergenic ORF (*orf245* at the *nad3/atp9* intergenic region) instead of two, and their *trn* genes showed variability in composition and order although tRNA molecules linking to all 20 amino acids were encoded in both species. *Myrothecium inundatum* differed from *S. chartarum* concerning the number of intergenic ORFs (3 vs. 2), the number (27 vs. 28) and order of *trn* genes, and the position of *cox1*. The *cox1* gene was located between *cob* and *nad1* in *Myr. inundatum*, whereas it was found between *rnl* and *nad2* in other Stachybotryaceae species.

It is also interesting to mention the joining pattern of two gene pairs, *nad2/nad3* and *nad4L/nad5* (Supplementary Table S1). The last nucleotide of the *nad2* termination codon (TAA) acted as the first nucleotide (A) of the *nad3* initiation codon (ATG) in *Mem. echinata* and *Myr. inundatum* (interval = −1), while *nad3* immediately followed *nad2* without nucleotide overlapping in *S. chartarum* and *S. chlorohalonata* (interval = 0). In all four Stachybotryaceae species, the *nad4L* termination codon (TAA) and the *nad5* initiation codon (ATG) overlapped by one nucleotide (interval = −1).

The four Stachybotryaceae species were conserved with regard to intron insertions except that an extra intron insertion was found in *S. chlorohalonata* (Supplementary Table S2). The *rnl* intron mL2450 and the *cox1* intron cox1P1125 were present in each species, plus corresponding intronic ORFs encoding Rps3 and a LAGLIDADG homing endonuclease. The extra intron in *S. chlorohalonata* also inserted into *cox1*, but this intron (cox1P212, group IB) encoded a GIY-YIG homing endonuclease. Sequences of either mL2450 or cox1P1125 showed high DNA sequence similarities (64–97%) among the four different species (Supplementary Table S3), suggesting their early localization into Stachybotryaceae and their dispersal during Stachybotryaceae speciation. The three introns were not homologous to one another. Online BLAST analyses, however, showed that each of the introns showed high similarity to introns in homologous genes from other fungi (Supplementary Table S4). This suggested that they might all originate from distantly related species by horizontal gene transfer.

### 2.4. Comparison of Mitogenomes among Different Species in Hypocreales

Since different Stachybotryaceae species displayed variations in gene arrangement patterns, we further investigated the gene order of all Hypocreales species with available mitogenomes (including 101 different species and 18 different varieties of *Fusarium oxysporum*), focusing on the order of 14 core PCGs and 2 rRNA genes (Supplementary Table S5). Two PCGs were absent in *Fusarium oxysporum* f. sp. *lactucae* (lacking *nad1* and *nad4*) and *Sarocladium implicatum* (lacking *cox3* and *nad6*), and all other species/varieties contained these 16 genes. The gene arrangement pattern of *Myr. inundatum* was dominant (109/119, designated as pattern A) among Hypocreales species and applied to 93 species and 16 *F. oxysporum* varieties. All species in Clavicipitaceae, Cordycipitaceae, Hypocreaceae, and Ophiocordycipitaceae and most species in Nectriaceae possessed pattern A. Different patterns, however, were seen in Bionectriaceae, Nectriaceae, Sarocladiaceae, Stachybotryaceae, and some family-undetermined species (Supplementary Table S5; Figure 2C). Specifically, except for the three Stachybotryaceae species whose *cox1* translocated to the *rnl*/*nad2* intergenic region (pattern B), the *nad4* gene in *S. implicatum* (the only Sarocladiaceae species with an available mitogenome) translocated to the *rnl*/*nad2* intergenic region (pattern C), too. *Cox2* in four Bionectriaceae/family-undetermined species (i.e., *Clonostachys rosea*, *Acremonium chrysogenum*, *A. fuci*, and *Emericellopsis* sp.) were found at the *nad4*/*atp8* intergenic region (pattern D) instead of the commonly found *atp9*/*nad4L* intergenic region. The order of *nad1* and *nad4* interchanged, and they were transcribed at the opposite strand in *F. oxysporum* f. sp. *matthiolae* (pattern E).

We further introduced *trn* genes into gene order comparisons of the above three patterns A, B, and D, each of which possessed more than one species (Supplementary Table S6). Our comparative study allowed the detection of three *trn* clusters, which situated downstream *rnl* (>10 *trn* genes), *rns* (3–5 *trn* genes), and *nad6* (4–5 *trn* genes). In addition, several regions were often inserted by a single *trn* gene, such as upstream *nad4L* (by *trnR*), downstream *cob* (by *trnC*), and downstream *cox3* (by *trnG*). For pattern B, accompanying the transposition of *cox1*, *trnP* was translocated from downstream *rns* to downstream *rnl*. Several *trn*-absent regions in pattern A became invaded by *trn* genes in patterns B (e.g., downstream *nad3*) and D (e.g., downstream *nad1*). Among species of each pattern, variations in *trn* gene positions could always be observed.

Since different Stachybotryaceae species displayed variations in their *nad2/nad3* joining patterns, we further investigated the *nad2/nad3* joining patterns in all the above Hypocreales species/varieties. A total of five different joining patterns for *nad2* and *nad3* were observed (Supplementary Table S5). In most Hypocreales mitogenomes (98/119), *nad3* immediately followed *nad2* without any nucleotide overlapping/separation (interval = 0). In most Cordycipitaceae species (15/16) and two out of the four Stachybotryaceae species (2/4), *nad2* and *nad3* overlapped by one nucleotide (interval = −1). In the remaining species, *nad2* and *nad3* were separated by one (in *C. rosea*), four (*Ophiocordyceps camponoti-floridani*), or six (*A. fuci* and *Emericellopsis* sp.) nucleotides. In addition, for all investigated Hypocreales species, the last nucleotide of the *nad4L* always acted as the first nucleotide of the *nad5* (interval = −1) without an exception. For every Hypocreales species, *rps3* was always present in *rnl* introns, which always inserted at a fixed position of *rnl*, viz. position 2450 relative to the *rnl* gene of *Escherichia coli* (AB035922).

### 2.5. Phylogenetic Analyses of Hypocreales Species

**Figure 3:**
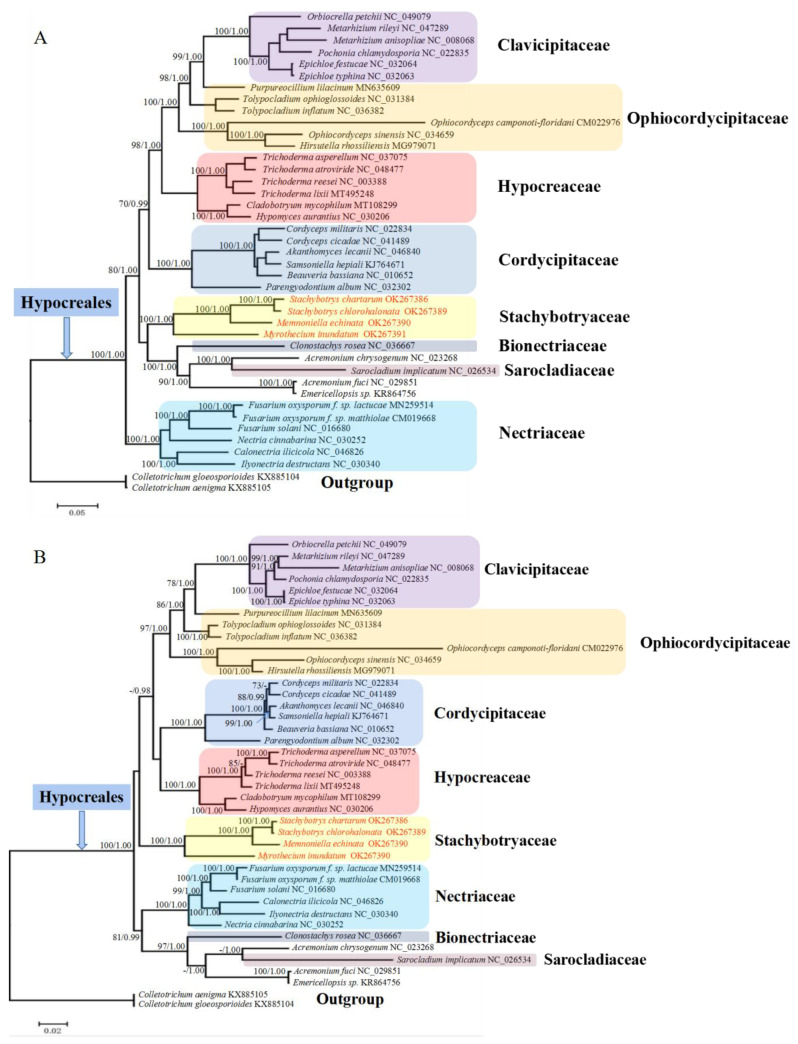
Phylogenetic analyses of Hypocreales species based on nucleotide (**A**) and amino acid (**B**) sequences of mitochondrial genes. Nucleotide and amino acid sequences (9446 and 3162 characters, respectively) of 14 standard mitochondrion-encoded genes (*atp6*, *atp8*, *atp9*, *cob*, *cox1–3*, *nad1–6* and *nad4L*) were used for phylogenetic analyses. The tree shown here was the single best topology recovered from ML, and the topology was identical to that recovered from BI. Support values from ML (before forward slash) and BI (after forward slash) analyses are given for nodes receiving strong supports (i.e., ML bootstrap values ≥70% or BI posterior probability ≥0.95). Members of each family are indicated in different colors, and those not shown in color were family-undetermined (i.e., Hypocreales incertae sedis).

Phylogenetic analyses were carried out using two different data sets (nucleotides and amino acids of 14 mitochondrion-encoded genes) and two different approaches (ML and BI). For each data set, both tree-building approaches generated identical topologies (Figure 3). The two different data sets showed only a subtle difference for familial relationships. Irrespective of the data sets and approaches, all Stachybotryaceae species always clustered as a separate clade with high support values (ML 100%, BI 1.00), and they might not be the most basal clade in Hypocreales. Species of Clavicipitaceae, Cordycipitaceae, Hypocreaceae, and Nectriaceae also each formed a separate clade. The only species in Sarocladiaceae (*S. implicatum*), the only species in Bionectriaceae (*C. rosea*), and three family-undetermined species (*A. chrysogenum*, *A. fuci*, and *Emericellopsis* sp.) formed a group. Species in Ophiocordycipitaceae failed to cluster together, and they scattered into three subclades. Among them, *Tolypocladium inflatum* and *T. ophioglossoides* formed a subclade. *Hirsutella rhossiliensis*, *Ophiocordyceps camponoti-floridani*, and *O. sinensis* formed another subclade. *Purpureocillium lilacinum*, as a single species in the third subclade, showed a sister relationship to Clavicipitaceae.

### 2.6. Ancestral State Estimation

**Figure 4:**
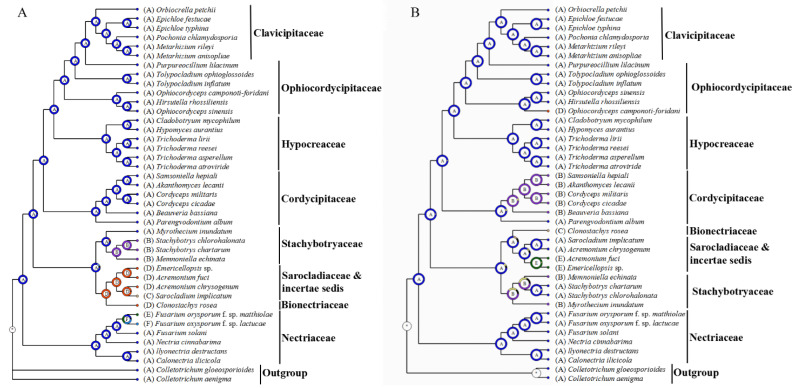
Inference of ancestral gene arrangement pattern (**A**) and ancestral *nad2/nad3* joining state (**B**). In panel A, each gene arrangement pattern is named by a different letter (A–F), which corresponds to the naming in Figure 2. In panel B, each *nad2/nad3* joining state is named by a different letter (A–E). A, no overlapping/separation (interval = 0); B, 1 bp overlapping (interval = −1); C, 1 bp separation (interval = 1); D, 4 bp separation (interval = 4); E, 6 bp separation (interval = 6).

Inference of ancestral gene arrangement patterns showed that the root node of Hypocreales had the dominant gene arrangement pattern (i.e., pattern A) with an occurrence of 99.3% (Figure 4A). The root node of Clavicipitaceae, Cordycipitaceae, Hypocreaceae, Nectriaceae, and the three subclades of Ophiocordycipitaceae all had the pattern A with an occurrence of >99.3%. Although the node consisting of the two *Stachybotrys* species and the root node of *Stachybotrys* and *Mem. echinata* had pattern B with an occurrence of >98.2%, the root node of Stachybotryaceae most likely had pattern A with an occurrence of >88.3%. The root node consisting of Sarocladiaceae/Bionectriaceae/family-undetermined species showed pattern D (occurrence 98%), while their common node to Stachybotryaceae showed pattern A (occurrence 81%). Patterns C, E, and F were each present in just one mitogenome. The above results indicate that pattern A was more ancestral than other patterns. Patterns B–F might all be derived from pattern A, and each of these gene rearrangement events occurred only once according to the phylogeny.

We also inferred the ancestral state of *nad2/nad3* joining patterns (Figure 4B). The root node of Hypocreales had the dominant state (i.e., no overlapping/separation, shown as state A) with an occurrence of 99.6%. The root node of Clavicipitaceae, Hypocreaceae, Nectriaceae, and the three subclades of Ophiocordycipitaceae all had state A with an occurrence of >99.9%. Although most species in Cordycipitaceae had state B (i.e., overlapping one nucleotide), the root node of Cordycipitaceae was inferred as state A with an occurrence of 93.2%. Although the node consisting of the two *Stachybotrys* species had state A with an occurrence of over 96.9%, the root node of Stachybotryaceae most likely had state B with an occurrence of 59.4%. The root node consisting of Sarocladiaceae/Bionectriaceae/family-undetermined species showed state A (occurrence 79.7%) although some species in this clade had state C (i.e., 1 bp separation) or E (i.e., 6 bp separation). These results indicate that *nad2/nad3* joining state A was more ancestral than other states. Events of transfer from state A to state B occurred at least twice, once within Cordycipitaceae and once during the speciation of Stachybotryaceae from their common ancestors. States C–E might all be derived from state A, and each of these states occurred only once according to the phylogeny.

### 2.7. Divergence Time Estimation

**Figure 5:**
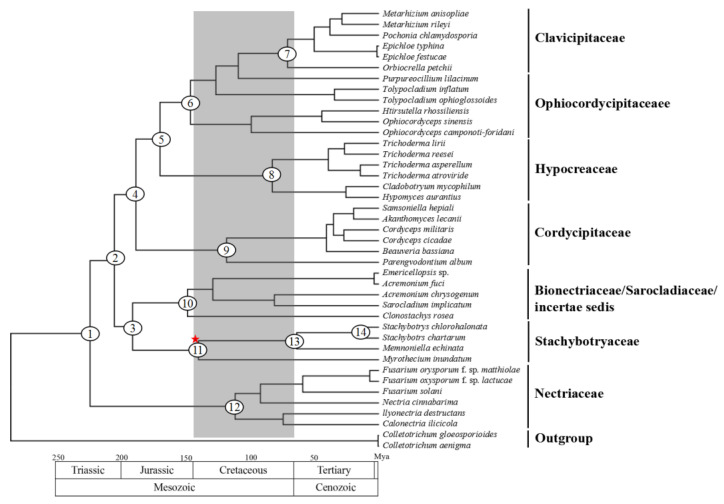
Divergence time estimation of fungal lineages in Hypocreales. Geological time is provided below the chronogram. Numbers in the circles correspond to some selected major nodes, of which the divergence times are shown in Table 2. Shaded area shows the divergence of major hypocrealean familial lineages. The node of Stachybotryaceae is marked with a red star.

Molecular dating was performed to estimate the divergence time of Stachybotryaceae. Two different time calibration priors (uniform and normal) generated almost identical results (Table 2). Dating analyses supported the supposition that the major hypocrealean familial lineages originated in the Jurassic and diversified in the Cretaceous (Figure 5), similar to those described in previous reports [17,18].The Stachybotryaceae family originated as an independent group in the Early Jurassic (192–194 Mya with 95% HPD 136–248, node 3) and diversified in the Early Cretaceous (141–142 Mya with 95% HPD 89–193, node 11). The genus *Stachybotrys* seemed to originate later than *Memnoniella* and *Myrothecium*.

## 3. Discussion

In this study, we reported for the first time complete mitogenomes of four Stachybotryaceae species, including *S. chartarum*, a species of type *Stachybotrys* and one of the world’s most feared fungi. The *S. chartarum* mitogenome had a size of 30.7 kb and contained genes typically found in fungal mitogenomes. A comparison of the mitogenomes of three different individuals of *S. chartarum* showed the conservation of gene contents/order and intron insertion positions as well as subtle variations in mitogenome sizes (30,718–30,745 bp). One of the individuals (IBT 7711) was recovered from Denmark, and the other two (IBT 40288 and 40293) were both recovered from the USA. The under-representation of isolates from broad regions might explain the observed low intraspecific diversity. For other fungi that have undergone intraspecific mitogenomic investigations, high intraspecific diversity was generally observed, with great intraspecific mitogenome size variations and intron presence/absence dynamics, such as *Cordyceps militaris* [19], *Hirsutella thompsonii* [8], and *Isaria cicadae* [5]. Low intraspecific diversity, however, was also reported in *Tolypocladium inflatum*, where no intron presence/absence dynamics were present among individuals from distantly separated localities [7]. It is possible that when more individuals of *S. chartarum* are sampled, a greater intraspecific diversity is seen.

Interspecific comparison of the mitogenomes of four different Stachybotryaceae species was further performed. The four Stachybotryaceae mitogenomes showed sizes of 28.7–31.4 kb. Among the 119 available mitogenomes of Hypocreales, only 20 mitogenomes had sizes smaller than 28.7 kb (Supplementary Table S5). Two introns (mL2450 and cox1P1125) were shared by these Stachybotryaceae mitogenomes, and identical intron-encoded proteins (Rps3 or LAGLIDADG homing endonuclease) were encoded by each intron from different species. Contrary to the low intraspecific genetic diversity in *S. chartarum*, different Stachybotryaceae species showed great mtDNA variations concerning intron insertion positions, gene order/contents, and *nad2/nad3* joining patterns (Supplementary Tables S1 and S2). As expected, *S. chartarum* was more similar to its congeneric species *S. chlorohalonata* than to the two other species in different genera in these respects. Number and length of introns, length of intergenic regions, and number of free-standing ORFs are all factors of mitogenome size variability in Stachybotryaceae (Table 1). Similar results are also reported in other fungal lineages [4,20].

Since comparisons on mitogenomes of different Stachybotryaceae species revealed variabilities in gene order and *nad2/nad3* joining pattern, we investigated all Hypocreales species with available mitogenomes. The over 100 mitogenomes of Hypocreales showed six different gene arrangement patterns and five different *nad2/nad3* joining patterns (Supplementary Table S5). A dominant pattern always existed in each case, and those rare patterns seemed to be confined to few fungal species or groups. Ancestral state estimation showed that the dominant gene arrangement pattern and the dominant *nad2/nad3* joining pattern were more ancestral than other patterns (Figure 4). No correlation could be found between gene arrangement patterns and *nad2/nad3* joining patterns. The addition of *trn* genes to the gene order comparison offered new insights into the above observed patterns. Some *trn* genes were always seen at fixed positions, while others appeared at variable positions among different patterns or even within a pattern (Supplementary Table S6). These *trn* genes in Hypocreales may play the role of hotspots for recombination and in extent of gene shuffling, similar to those reported in metazoan and other fungi [21,22]. When comparing fungal mitogenomes at a broader species sampling, there are even higher variabilities in gene order [6,23]. Each of the *nad2/nad3* joining patterns (overlapping or directly following) was also reported in other fungi [24,25].

Despite the above variabilities, different Hypocreales mitogenomes showed some conserved characteristics (Supplementary Table S5). For example, all Hypocreales mitogenomes contained 14 core PCGs and 2 rRNA genes with two exceptions (*Fusarium oxysporum* f. sp. *lactucae* and *Sarocladium implicatum*). The joining pattern of *nad4L* and *nad5* (i.e., one base overlapping) was conserved among all Hypocreales mitogenomes. The *rps3* gene in Hypocreales was always present in an *rnl* intron (i.e., mL2450). In non-Hypocreales mitogenomes, *rps3* can be intron-encoded (within mL2450), free-standing (i.e., encoded within intergenic regions), or missing (likely complemented by a nuclear-encoded analog) [26,27]. The universal existence of both mL2450 and *rps3* in Hypocreales suggests an origin from a common ancestor through horizontal gene transfer. The possible coevolution of mL2450 and *rps3* is also anticipated since fungal mitochondrial introns and their homing endonucleases (GIY-YIG and LAGLIDADG) have been reported to be coevolved [28].

Phylogenetic analyses were performed based on mitochondrion-encoded genes in this study, and they all supported the grouping together of different Stachybotryaceae species and the placement of Stachybotryaceae in the order of Hypocreales (Figure 3). This is consistent with previous studies deduced from nuclear genes [10]. According to our phylogenetic analyses, members of Clavicipitaceae, Cordycipitaceae, Hypocreaceae, and Nectriaceae can each group together, while members of Ophiocordycipitaceae scattered into three separate subclades (Figure 3). The inability of Ophiocordycipitaceae species to group together was also reported in some other studies that used mitochondrial sequences for phylogenetic reconstructions [7,18]. One study reports that excluding *atp6* from the concatenated data set ensures the clustering of Ophiocordycipitaceae because phylogenetic conflicts are present between *atp6* and other genes [8]. A partition homogeneity test was performed for our datasets, and conflicts were detected between most mitochondrial gene pairs (Supplementary Table S7). There are studies showing that even if different genes have phylogenetic conflict, they could also be combined together [29]. All the 14 mitochondrial genes/proteins in our dataset were therefore concatenated for phylogenetic analyses because mitochondrial genes are deemed to have a single history. The exact reason why species of Ophiocordycipitaceae cannot group together based on mitochondrion-encoded genes remains to be determined. In addition, *Sarocladium implicatum* (syn. *Acremonium implicatum*), which is currently still recorded as Hypocreales insertae sedis in Index Fungorum (http://www.indexfungorum.org/, accessed on 27 September 2021), was classified into Sarocladiaceae in a recent study [30]. *Acremonium fuci* and *Emericellopsis* sp. clustered together with very short genetic distance in our analyses. These two species may be classified into the recently established family Myrotheciomycetaceae, which has included the genus *Emericellopsis* [31]. Obviously, species of Hypocreales incertae sedis should be broadly sampled in the future to finally resolve their taxonomy. At least, the two *Acremonium* species (*A. chrysogenum* and *A. fuci*) most likely not belong to the same genus because of a deep phylogenetic separation (Figure 3).

Molecular dating analysis showed that the crown age of Stachybotryaceae was inferred to be approximately the Early Cretaceous (141–142 Mya) (Table 2; Figure 5). This estimated age is younger than the 176 Mya reported previously [17], probably because fewer and different samples were included in our study or because different gene markers (mitochondrial vs. nuclear) were used. A nuclear dataset is expected to present different substitution/evolution rates compared to a mitochondrial dataset [32]. We further found that the genus *Stachybotrys* originated later than the other two genera *Memnoniella* and *Myrothecium* in Stachybotryaceae (Figure 5). This result is consistent with that reported previously [17]. Different from the study of Sung et al. (2008), where the *Stachybotrys* clade (so called because the family Stachybotryaceae was not established by that time) was resolved as a basal lineage of Hypocreales, our current study found that Nectriaceae seemed to be more basal than other families in Hypocreales.

In conclusion, this study reported six mitogenomes of four different Stachybotryaceae species. Mitogenomes of different individuals of *S. chartarum* were rather conserved with low intraspecific diversity. Mitogenomes of different Stachybotryaceae species, however, showed variabilities in intron insertion positions, gene order/contents, and *nad2/nad3* joining patterns. Further investigations on all Hypocreales species with available mitogenomes showed greater variability in gene order (six different patterns) and *nad2/nad3* joining pattern (five different patterns), with the dominant patterns being more ancestral than other patterns in each case. Phylogenetic analyses based on 14 mitochondrion-encoded genes supported the placement of Stachybotryaceae in Hypocreales, and the crown age of Stachybotryaceae was inferred to be approximately the Early Cretaceous (141–142 Mya).

## 4. Materials and Methods

### 4.1. Assembly and Annotation of Mitogenomes

Clean reads resulting from Illumina sequencing were downloaded online from the SRA database (https://www.ncbi.nlm.nih.gov/home/download/, accessed on 22 February 2021). These included data for three individuals of *S. chartarum* and one individual each of *S. chlorohalonata*, *Mem. echinata*, and *Myr. inundatum* (Table 1). Those of *S. chartarum* and *S. chlorohalonata* were sequenced by Semeiks et al. (2014) [15]. Those of *Mem. echinate* (sequenced by RIKEN Center for Life Science Technologies, Division of Genomic Technologies, Japan) and *Myr. inundatum* (by DOE Joint Genome Institute, USA) were not published in the literature. Mitogenomes were assembled de novo using programs NOVOPlasty v4.3.1 [33] and GetOrganelle v1.7.5 [34]. Assembled mitogenomes were annotated as described previously [7]. Putative open reading frames (ORFs) ≥300 nt at intronic and intergenic regions were recognized in this study, and they were named with “orf” and length of encoded proteins. Introns in *rnl* and protein-coding genes were determined and named according to the established nomenclatures [35,36].

### 4.2. Comparison among Different Mitogenomes

We performed mitogenome comparisons not only among different individuals of *Stachybotrys chartarum* but also among different species of Stachybotryaceae and Hypocreales. All mitogenome sequences were adjusted to start from *rnl*, and multiple sequence alignments were performed using MAFFT v7.453 [37]. Nucleotide variations were determined using DnaSP v6.12.03 [38]. A comparative map was generated using Mauve v20150226 [39] to visualize if there were syntenies among different mitogenomes. Alignment and graphical visualization of mitogenomes were also carried out by BRIG v0.95 [40]. All Hypocreales species with available mitogenomes were examined to investigate their gene order of core PCGs and rRNA genes as well as their *nad2/nad3* joining patterns.

### 4.3. Phylogenetic Tree Reconstruction

To determine the phylogenetic position of Stachybotryaceae in Hypocreales, we performed phylogenetic analyses using two different datasets: nucleotide sequences and amino acid sequences of 14 standard mitochondrion-encoded genes (*atp6*, *atp8*, *atp9*, *cob*, *cox1–3*, *nad1–6* and *nad4L*). Representative species from all families of Hypocreales with available mitogenomes were chosen as ingroups (Supplementary Table S8). Two Glomerellales species (*Colletotrichum aenigma* and *Colletotrichum gloeosporioides*) were employed as outgroups. For each data set, best-fit partitioning schemes and models of evolution for each subset (Supplementary Table S9) were determined according to PartitionFinder v2.1.1 [41]. Phylogenetic relationships were inferred using both Bayesian inference (BI) and maximum likelihood (ML) approaches. Specifically, BI phylogenetic analysis was performed using MrBayes v3.2.7 [42]. Two simultaneous MCMC (Markov Chain Monte Carlo) runs with four chains each (3 hot and 1 cold) were performed for one million generations and sampled every 100 generations, discarding a burn-in of 20%. ML topology searches were completed with IQ-TREE v1.6.12 with 1000 bootstrap replicates [43]. We considered one branch to be strongly supported if it received posterior probability ≥0.95 (for BI) or bootstrap values ≥70% (for ML).

### 4.4. Ancestral State Inference

To speculate on the ancestral gene arrangement pattern and the ancestral *nad2/nad3* joining state, the nexus-formatted .t file and the .con.tre file generated from the above nucleotide BI analysis was used to perform a Bayesian Binary MCMC (BBM) analysis in RASP v4.2 [44]. Corresponding gene arrangement patterns (designated as letters A–F) or *nad2/nad3* joining states (designated as letters A–E) were assigned to each species. The analyses were run under the best available model (Supplementary Table S9) across 10 Markov chains (500 thousand generations each, with trees sampled every hundred generations) discarding the first 1000 trees as burn-in.

### 4.5. Divergence Time Estimation

To speculate on the divergence time of Stachybotryaceae, the same nucleotide data set used above for phylogenetic analyses was employed for a Bayesian inference in BEAST v2.6.6 [45]. Calibration priors of the crown clade of Hypocreales, with reference to Sung et al., 2008, were set by two different ways, a uniform prior between 158 Mya and 232 Mya and a normal prior with a mean value of 193 Mya and a Sigma value of 23.5 Mya. Partitions and site models (Supplementary Table S9) were determined according to PartitionFinder v2.1.1 [41]. Trees and clock models were linked, but site models were unlinked across different partitions. A Relaxed Clock Log Normal molecular clock model was applied with the Calibrated Yule Model as the tree prior. For each calibration prior, two MCMC analyses were run for 100 million generations with parameters sampled every 1000 generations. The output of BEAST was analyzed using Tracer v1.7.2 [46] to make sure that effective sample sizes (ESSs) for all parameters were well above 300. Tree files generated by the two separate runs of each calibration prior were combined using LogCombiner v2.6.6 [45]. The combined tree file was used by TreeAnnotator v2.6.6 [45] to identify a best supported tree, to calculate the posterior clade probability for each node, and to annotate this selected tree topology with the mean ages of all the nodes as well as the 95% highest posterior density (HPD) interval of divergence times for each clade in the selected tree. The chronogram was finally visualized using FigTree v1.4.4 (http://tree.bio.ed.ac.uk/software/figtree/, accessed on 25 September 2021).

## Figures and Tables

**Table 1:** Summary of different mitogenomes of Stachybotryaceae.

Item	IBT 7711	IBT 40288	IBT 40293	IBT 40285	JCM 22618	CBS 120646
Species	*Stachybotrys chartarum*	*Stachybotrys chartarum*	*Stachybotrys chartarum*	*Stachybotrys chlorohalonata*	*Memnoniella echinata*	*Myrothecium inundatum*
Geography	Denmark	Oakland CA, USA	Oakland CA, USA	Oakland CA, USA	--	--
Isolation source	Building materials	Building materials	Building materials	Building materials	Unknown	Unknown
Run accession	SRR628249	SRR628247	SRR628248	SRR628246	DRR032509	SRR3439795
Mitogenome size (bp)	30,745	30,718	30,745	31,375	28,743	30,041
Accession no.	OK267386	OK267387	OK267388	OK267389	OK267390	OK267391
AT%	73.7	73.7	73.7	73.8	74.5	73.2
AT skew	−0.02	−0.02	−0.02	−0.01	−0.02	0.00
GC skew	0.12	0.12	0.12	0.12	0.15	0.13
No. standard PCGs	14	14	14	14	14	14
No. free-standing ORFs	2	2	2	2	1	3
No. rRNAs	2	2	2	2	2	2
No. tRNAs	28	28	28	28	28	27
No. introns	2	2	2	3	2	2
No. intronic ORFs	2	2	2	3	2	2
Intronic region (nt) ^†^	3884	3884	3884	5123	3905	4029
Intron-containing genes	*rnl*; *cox1*	*rnl*; *cox1*	*rnl*; *cox1*	*rnl*; *cox1*	*rnl*; *cox1*	*rnl*; *cox1*
Genic region (nt) ^‡^	25,239	25,230	25,239	26,410	24,526	25,700
Genic region (%)	82.1	82.1	82.1	84.2	85.3	85.6
Intergenic regions ^‡^ (nt)	5506	5488	5506	4965	4217	4341
Intergenic regions (%)	17.9	17.9	17.9	15.8	14.7	14.4

^†^ The length of intronic regions included that of intronic ORFs in this table. ^‡^ The length of genic regions considered intronic regions because introns were harbored by genes. Intergenic regions were those among PCGs, rRNA, tRNAs, and free-standing ORFs.

**Table 2:** Bayesian estimates of divergence times (Mya), including 95% highest posterior density (HPD) for major phylogenetic nodes ^†^.

Node	Age (95% HPD)		Note
	Normal Prior	Uniform Prior	
1	225 (160, 293)	228 (172, 289)	
2	206 (149, 269)	208 (159, 264)	
3	192 (136, 248)	194 (146, 245)	
4	189 (133, 246)	191 (144, 243)	
5	170 (118, 223)	172 (127, 223)	
6	147 (102, 194)	148 (106, 194)	Clavicipitaceae/Ophiocordycipitaceae
7	71 (44, 100)	72 (45, 101)	Clavicipitaceae
8	83 (46, 125)	88 (49, 132)	Hypocreaceae
9	119 (71, 171)	119 (73, 169)	Cordycipitaceae
10	149 (100, 200)	149 (105, 199)	Sarocladiaceae/Bionectriaceae/incertaesedis
11	141 (89, 193)	142 (94, 193)	Stachybotryaceae
12	112 (67, 159)	120 (74, 169)	Nectriaceae
13	64 (32, 100)	65 (33, 99)	
14	11 (4, 20)	12 (4, 20)	*Stachybotrys*

^†^ Two time calibration priors (normal and uniform distributions) were used, and they generated similar results. Fungal lineages represented by some nodes of interest are noted.

## Data Availability

The mitogenome sequences newly generated in this study were submitted to GenBank under accession numbers OK267386–OK267391.

## Supplementary Materials

The following are available online at https://www.mdpi.com/article/10.3390/ijms222413341/s1.
