# Supplementary material for: Comparative Mitogenomics of Fungal Species in Stachybotryaceae Provides Evolutionary Insights into Hypocreales
Source: Int J Mol Sci. 2021 Dec 12;22(24):13341. doi: 10.3390/ijms222413341 (PMC8706829; doi:10.3390/ijms222413341)
Supplement: Supplementary file 1 [file ijms-22-13341-s001.zip › Supporting/Supplementary Figures.pptx]

## Slide 1
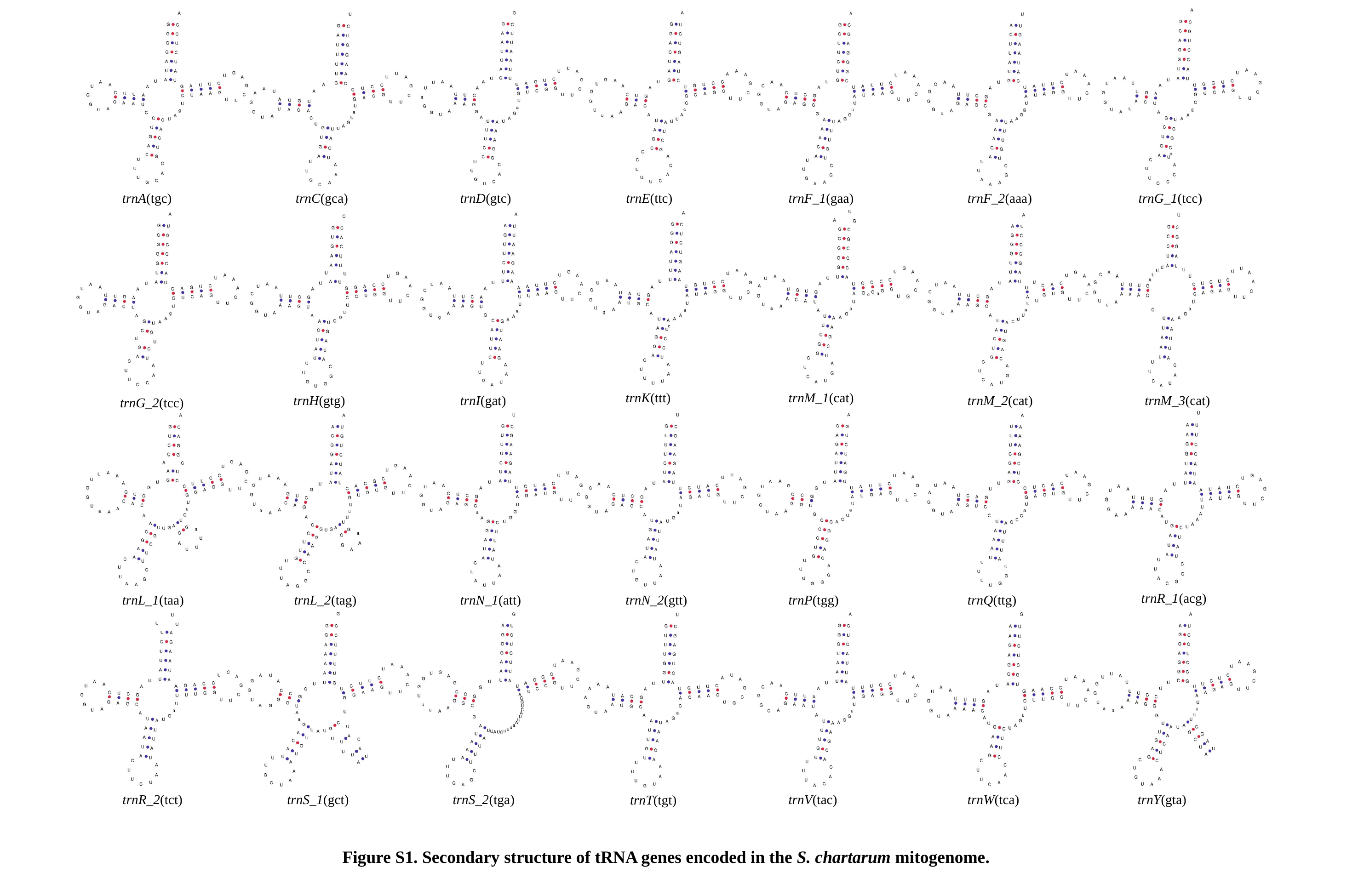

trnE(ttc)
trnD(gtc)
trnA(tgc)
trnF_1(gaa)
trnC(gca)
trnF_2(aaa)
trnG_1(tcc)
trnM_1(cat)
trnK(ttt)
trnH(gtg)
trnM_2(cat)
trnM_3(cat)
trnI(gat)
trnG_2(tcc)
trnR_1(acg)
trnL_1(taa)
trnL_2(tag)
trnN_1(att)
trnN_2(gtt)
trnP(tgg)
trnQ(ttg)
trnR_2(tct)
trnS_1(gct)
trnS_2(tga)
trnV(tac)
trnW(tca)
trnY(gta)
trnT(tgt)
Figure S1. Secondary structure of tRNA genes encoded in the S. chartarum mitogenome.
